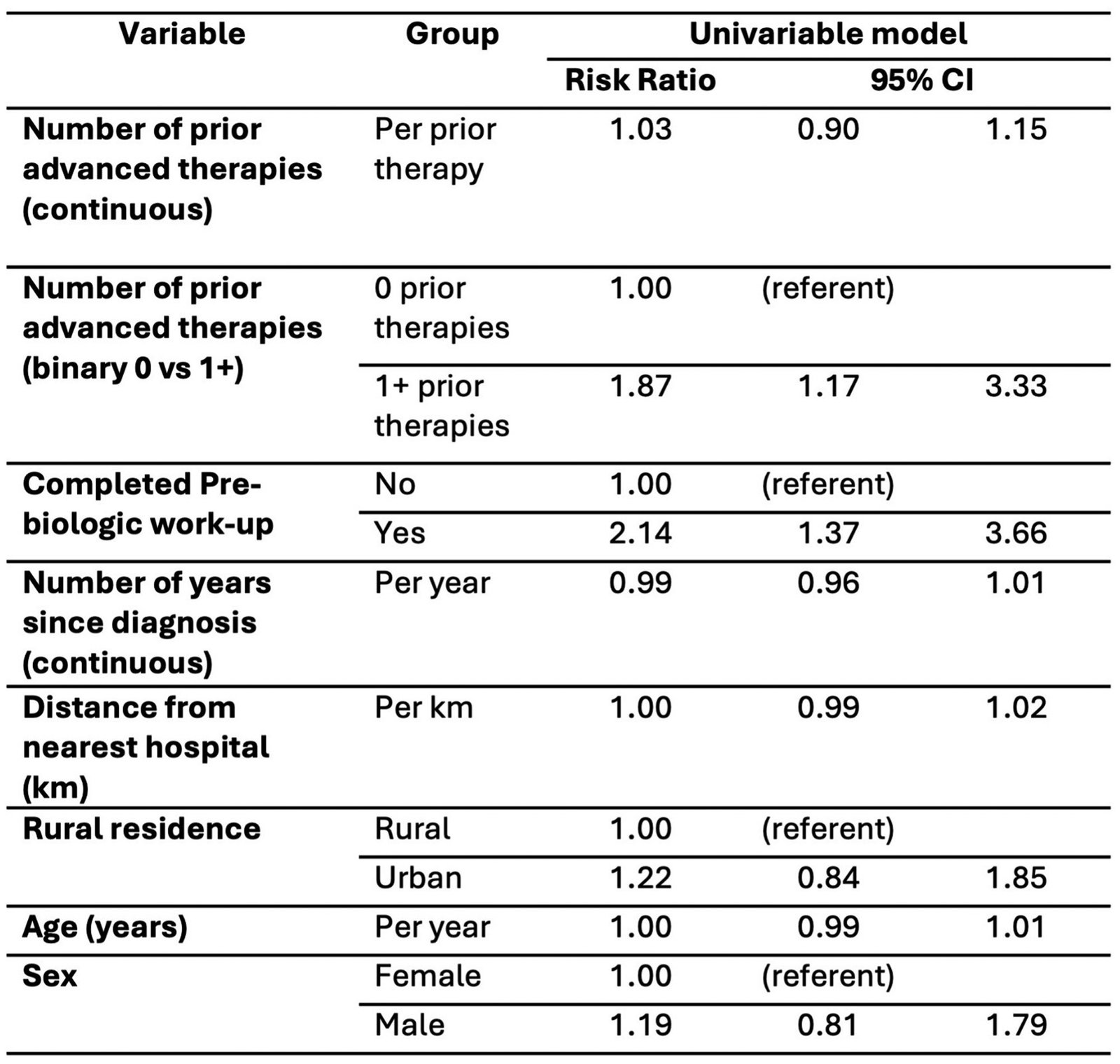# Poster Session II - A310 EVALUATION OF BARRIERS TO OUTPATIENT ADVANCED THERAPY INITIATION IN PATIENTS WITH INFLAMMATORY BOWEL DISEASE

**DOI:** 10.1093/jcag/gwaf042.310

**Published:** 2026-02-13

**Authors:** P Shelemey, P Dhingra, L M van Lierop, F Hoentjen

**Affiliations:** Gastroenterology, University of Alberta Faculty of Medicine & Dentistry, Edmonton, AB, Canada; University of Alberta Faculty of Medicine & Dentistry, Edmonton, AB, Canada; Gastroenterology, University of Alberta Faculty of Medicine & Dentistry, Edmonton, AB, Canada; Gastroenterology, University of Alberta Faculty of Medicine & Dentistry, Edmonton, AB, Canada

## Abstract

**Background:**

STRIDE-II guidelines recommend early initiation of advanced therapy in inflammatory bowel disease (IBD) following a treat-to-target approach in order to achieve deep remission and prevent complications. Despite these guidelines, 50% of patients with Crohn’s disease (CD), and 40% of patients with ulcerative colitis (UC) have suboptimal disease control and delayed therapy initiation is common.

**Aims:**

To determine the time from decision to initiation of advanced IBD therapy in the outpatient setting and to identify factors associated with initiation in less than 30 days.

**Methods:**

In this single-center retrospective cohort study at a high-volume University Hospital, we included adult IBD patients with a decision made to start advanced therapy, regardless of prior exposure, as an outpatient between September 2023 – February 2024. Inpatient therapy starts were excluded. The primary outcome was the proportion of patients achieving advanced therapy initiation within 30 days of decision to initiate. Associations with initiating advanced therapy in less than 30 days were assessed by log-binomial regression for age, sex, prior advanced therapies, pre-biologic work-up completion, disease duration, distance to nearest hospital, and urban/rural residence.

**Results:**

We included 132 patients; mean age was 45 and 57.6% of patients were male. 69.7% had CD, 28% had UC, and 2.3% had unclassified IBD. Overall, 45% of patients initiated advanced therapy within 30 days (mean 18 ± 8.4), and 55% after 30 days (mean 69 ± 51). The log-binomial regression analysis showed that having an up-to-date pre-biologic work up at time of decision to initiate advanced therapy increased the likelihood of advanced therapy initiation in less than 30 days (RR 2.14; 95% CI 1.37, 3.66), as did exposure to prior advanced therapy (RR 1.87; 95% CI 1.17, 1.35). Disease duration, rural zip code, distance to nearest hospital, and type of advanced therapy, were not associated with therapy initiation timelines.

**Conclusions:**

The majority of IBD patients starting advanced therapy required greater than 30 days to initiate. Completion of pre-biologic workup and prior advanced therapy exposure were associated with earlier initiation. Future directions include implementing interventions such as optimized patient education resources and early, streamlined pre-biologic testing, aiming to shorten time to advanced therapy initiation and improve disease control.

Log-binomial regression analysis presenting a series of univariate models for the association between select variables and initiation of advanced therapy within 30 days from decision to initiate.

**Funding Agencies:**

None